# Moral Judgements on the Actions of Self-Driving Cars and Human Drivers in Dilemma Situations From Different Perspectives

**DOI:** 10.3389/fpsyg.2019.02415

**Published:** 2019-11-01

**Authors:** Noa Kallioinen, Maria Pershina, Jannik Zeiser, Farbod Nosrat Nezami, Gordon Pipa, Achim Stephan, Peter König

**Affiliations:** ^1^Institute of Cognitive Science, Osnabrück University, Osnabrück, Germany; ^2^Institute of Philosophy, Leibniz University Hannover, Hanover, Germany; ^3^Department of Neurophysiology and Pathophysiology, Center of Experimental Medicine, University Medical Center Hamburg-Eppendorf, Hamburg, Germany

**Keywords:** self-driving cars, moral judgement, ethics, virtual reality, moral dilemmas, autonomous vehicles, artificial intelligence ethics

## Abstract

Self-driving cars have the potential to greatly improve public safety. However, their introduction onto public roads must overcome both ethical and technical challenges. To further understand the ethical issues of introducing self-driving cars, we conducted two moral judgement studies investigating potential differences in the moral norms applied to human drivers and self-driving cars. In the experiments, participants made judgements on a series of dilemma situations involving human drivers or self-driving cars. We manipulated which perspective situations were presented from in order to ascertain the effect of perspective on moral judgements. Two main findings were apparent from the results of the experiments. First, human drivers and self-driving cars were largely judged similarly. However, there was a stronger tendency to prefer self-driving cars to act in ways to minimize harm, compared to human drivers. Second, there was an indication that perspective influences judgements in some situations. Specifically, when considering situations from the perspective of a pedestrian, people preferred actions that would endanger car occupants instead of themselves. However, they did not show such a self-preservation tendency when the alternative was to endanger other pedestrians to save themselves. This effect was more prevalent for judgements on human drivers than self-driving cars. Overall, the results extend and agree with previous research, again contradicting existing ethical guidelines for self-driving car decision making and highlighting the difficulties with adapting public opinion to decision making algorithms.

## 1. Introduction

Self-driving cars are rapidly becoming a reality. In 2016, car manufacturer Tesla announced that all of its current cars were being equipped with the hardware necessary for autonomous driving (The Tesla Team, 2016). Since then, Tesla has incrementally enabled autonomous and assisted driving features via software updates (The Tesla Team, 2019). Other manufacturers have since been following suit (see Mercer and Macaulay, 2019) and the use of partially self-driving cars, such as these, is expected to increase within the next 20 years.

A major argument supporting the development of self-driving cars is the expected reduction in the number of traffic accidents. For example, close to 90% of the more than 300,000 traffic accidents resulting in injuries to people in Germany in 2017 were caused by driver misconduct or error, such as ignoring right of way, inappropriate following distance or speed, overtaking faults, and driving under the influence of alcohol (Statistisches Bundesamt, 2018, p. 49). Similar observations have been made in both the United Kingdom and the United States (National Highway Traffic Safety Administration, 2008; Department for Transport, 2013). These errors and misconduct can potentially be mitigated by the introduction of self-driving cars, which highlights their potential to improve public safety.

However, the expected reduction of accidents will need time to be realized. Recently published statistics by the California Department of Motor Vehicles shows that self-driving car prototypes are involved in accidents at a similar rate as human drivers (Favarò et al., 2017). Other reports give somewhat more favorable numbers with a reduction of accident rates by about one third (Marshall, 2018; Thomas, 2018). The discrepancy to the optimistic forecasts cited above stems in part from an increase of, for example, unexpected breaking resulting in rear-end collisions, and the fact that even when an accident is not caused by a self-driving car, it might still be involved in it. Thus, during a multi-year introduction period, self-driving cars will be involved in a substantial number of accidents and unexpected situations.

Unexpected traffic situations are often highly complex and require split-second decisions. For this reason, human drivers are not generally expected to be able to respond optimally and may be excused for making wrong decisions (Trappl, 2016). Self-driving car control systems, on the other hand, can potentially estimate the outcome of various options within milliseconds and take actions that factor in an extensive body of research, debate, and legislation (Lin, 2015). The actions taken in such situations have potentially harmful consequences for car occupants, other traffic participants, and pedestrians. Therefore, it is important to carefully consider the ethics of how self-driving cars will be designed to make decisions, an issue that is the topic of current debate (Nyholm, 2018a,b; Dietrich and Weisswange, 2019; Keeling et al., 2019).

Comprehensive guidelines for ethical decision making for self-driving cars have been provided by the ethics commission of the German Federal Ministry of Transport and Digital Infrastructure (2017). These guidelines speak out against a standardized procedure of decision making in dilemma situations (guideline 8). In cases of unavoidable accidents, “any distinction based on personal features (age, gender, physical, or mental constitution) is strictly prohibited” and “[those] parties involved in the generation of mobility risks must not sacrifice non-involved parties” (guideline 9). These guidelines greatly add to the discussion and can inform the development of decision making systems. However, it is far from obvious that a practical implementation of these guidelines would garner public consensus.

As pointed out by Shariff et al. (2017), and further evident by the number of studies focusing on public opinion (see Gkartzonikas and Gkritza, 2019, for a review) the introduction of self-driving cars requires acceptance from the public. Empirical research investigating public perception and beliefs can be useful for highlighting areas problematic for the acceptance of self-driving cars into public traffic. Such research in the area of ethical decision making for self-driving cars has primarily focused on human decision making as a basis. In a typical experiment, participants make decisions pertaining to hypothetical dilemma situations in which harm is unavoidable. Situations of this kind, known as trolley dilemmas (Thomson, 1985), involve two groups of people, one of which must be endangered to spare the other. The utility of trolley dilemmas does not lie in their use as blueprints for crash optimizations (Holstein and Dodig-Crnkovic, 2018). Rather, they are an effective means to elucidate which ethical values are potentially conflicting in accident scenarios and to allow for the design of self-driving cars informed by human values (Gerdes et al., 2019; Keeling, 2019). As argued by Bonnefon et al. (2019), trolley dilemmas should not be understood primarily as simulations of real-life scenarios, but as representations of conflicts that emerge on a statistical level: the introduction of self-driving cars will likely put different people at risk compared to today. For example, would it be acceptable that due to self-driving cars, fewer people are harmed in traffic, but those who are harmed are more likely to be pedestrians than car occupants?

Moral dilemma studies can be grouped broadly into two paradigms: those that investigate moral judgements (what people claim are the right actions) and those that investigate moral actions (what people actually do in given situations). An analysis of more than 40 million judgements on vignettes describing hypothetical dilemma situations concluded that people generally prefer self-driving cars to endanger fewer lives, endanger animals over people and endanger older people over younger people (Awad et al., 2018). Other moral judgement studies include simulation studies by Wintersberger et al. (2017) and Wilson et al. (2019) and vignette-based studies by Bonnefon et al. (2016), Li et al. (2016), Meder et al. (2018), Smith (2019), and Rhim et al. (2020). Importantly, Bonnefon et al. (2016) found a discrepancy between what people deemed acceptable for self-driving cars to do in dilemma situations and their willingness to purchase cars that would act accordingly. Specifically, people considered it more morally acceptable for self-driving cars to endanger fewer lives, even at the expense of the occupants' lives, but preferred to purchase cars that would protect occupants. Martin et al. (2017) suggested that this discrepancy may be resolved if people explicitly consider the situations from both the perspectives of car occupants and pedestrians. Borenstein et al. (2019) highlighted that the perspectives of pedestrians and other non-occupants is overshadowed by the focus on car occupants in the literature, but are equally important.

Studies of moral action have used virtual reality environments to determine how human drivers would act when faced with dilemma situations. In these studies, participants were put in the perspective of drivers and controlled the steering of virtual vehicles when facing such dilemma situations. Skulmowski et al. (2014) placed participants in the role of train drivers and found participants generally preferred to save the greater number of lives. Sütfeld et al. (2017) found that the behavior of participants in the role of car drivers could be well described by a value-of-life model, such that people are valued more than animals and younger people are valued more than older. Faulhaber et al. (2018) (further elaborated by Bergmann et al., 2018), Li et al. (2019) showed that car drivers also tend to act in ways that endanger fewer lives, even at the expense of their own. Ju et al. (2019) found that personality characteristics predict the likelihood of drivers endangering themselves. Furthermore, Luzuriaga et al. (2019) directly compared actions chosen by participants tasked with programming a self-driving car with actions made by participants in a driving simulator. They found that participants programming a self-driving car more readily endangered car occupants to save pedestrians, than participants driving in a simulator. Thus, our knowledge of how humans act in critical situations in virtual reality is increasing.

While the results of these moral judgement and moral action studies have been generally consistent, there are important distinctions between the approaches needing consideration before making strong conclusions. First, there is growing evidence of discrepancies between what people consider to be the right action in moral dilemmas and what they would actually do (e.g., FeldmanHall et al., 2012; Tassy et al., 2013; Patil et al., 2014; Gold et al., 2015; Francis et al., 2016). Additionally, what is generally considered ethical for human drivers may not be the same for self-driving cars. Furthermore, the perspective from which the situations are presented may affect how they are evaluated.

To address aforementioned issues, we conducted two studies in the moral judgements paradigm which allowed us to investigate moral beliefs about self-driving cars and human drivers in dilemmas situations from different perspectives. In both studies, we recorded judgements pertaining to virtual dilemma situations involving either self-driving cars or human drivers. We included the perspectives of car occupants, uninvolved observers and pedestrians, which to our knowledge, no previous studies have done. Study 1 employed virtual reality to investigate judgements in specific dilemma situations, while Study 2 used simplified animations and varied aspects of the situations in a more fine-grained manner.

## 2. Study 1—Moral Judgements in Virtual Reality

In this study, we addressed the effects of perspective (passenger, pedestrian, or observer) and type of motorist (human driver or self-driving car) on moral judgements in immersive virtual environments. We investigated three different scenarios, all involving the choice between endangering one of two groups of virtual avatars. The scenarios were designed to be morally ambiguous to avoid ceiling or floor effects. We hypothesized a self-preservation effect, such that, independent of the type of motorist, participants would be less likely to judge actions that endangered their own virtual avatars as more acceptable.

### 2.1. Materials and Methods

#### 2.1.1. Participants

One hundred and eighty-four people (96 male, 88 female) voluntarily participated in the virtual reality experiment. Participants were recruited through social media, university mailing lists, word of mouth, or were directly approached. Participants could earn experiment participation credits required for some university programs, but no monetary incentive was provided. Participants were required to be at least 18 years old with native-level German and gave written informed consent after being briefed on the content of the experiment. Exclusion criteria included having experienced previous car-related trauma, being prone to motion sickness and having a history of epileptic seizures. The study was approved by the ethics review board at Osnabrück University, Germany. Descriptive statistics of the participants are shown in Table S1.

#### 2.1.2. Materials

The stimuli consisted of six pairs of virtual reality animations, each approximately 30 s in duration, created with Unity (Unity Technologies, 2018). Each scenario involved a car with two occupants: driver and passenger (human driver condition) or two passengers (self-driving car condition). The car drove in the middle of a road and encountered a dilemma situation in which it could veer either to the left or the right, endangering one of two groups of avatars. Animations depicting both possible actions were shown in sequence.

To prevent unnecessary distress, the animations and sound effects in the virtual environment ceased immediately before the car would be involved in a collision. A braking sound effect was played in the moments before the animations ended to demonstrate that the car attempted, but was unable, to stop before impact. Participants had no control over the car or avatars, but could freely observe the virtual environment. If the motorist was a self-driving car, the steering wheel of the car was absent and a label was shown at the front of the car indicating that it was self-driving in order to remind participants during the course of the experiment. Three different scenarios were investigated: child pedestrians vs. adult pedestrians; pedestrians on the road vs. pedestrians on the sidewalk; and car occupants vs. pedestrians. Each scenario included two different trials.

In the child pedestrians vs. adult pedestrians scenario the car either veered toward a group of pedestrians including children or a group of only adult pedestrians. The two trials differed by group size, but the ratio was static. In the smaller groups trial, there was one child (and an adult viewpoint avatar) in one group and two adults (and an adult viewpoint avatar) in the other group; in the larger groups trial, there were two children (and an adult viewpoint avatar) in one group and four adults (and an adult viewpoint avatar) in the other group.

In the pedestrians on the road vs. pedestrians on the sidewalk scenario, the car veered toward either adult pedestrians standing on the sidewalk or adult pedestrians standing on the road. The two trials differed by group size, but the ratio was static. In the smaller groups trial, there was one pedestrian on the sidewalk and two pedestrians on the road; in the larger groups trial, there were two pedestrians on the sidewalk and four on the road.

In the car occupants vs. pedestrians scenario, the car veered toward either the pedestrians on the road or an obstacle that would endanger the lives of the car occupants. Instead of varying by the size of the groups, the two trials differed by the type of obstacle. In the parked van trial, the car would veer toward a large van parked on the side of the road, whereas in the cliff trial, the car would veer toward a cliff edge. Both variations of these scenarios are equivalent in the implied outcome: either car occupants or pedestrians will be harmed. While Faulhaber et al. (2018) only investigated endangering car occupants in the context of a cliff setting, we wanted to contrast this scenario with a less extreme setting. By having the car veer toward a parked van, harm toward car occupants is still implied, but the scenario is overall more integrated into a typical traffic setting.

**Figure 1:**
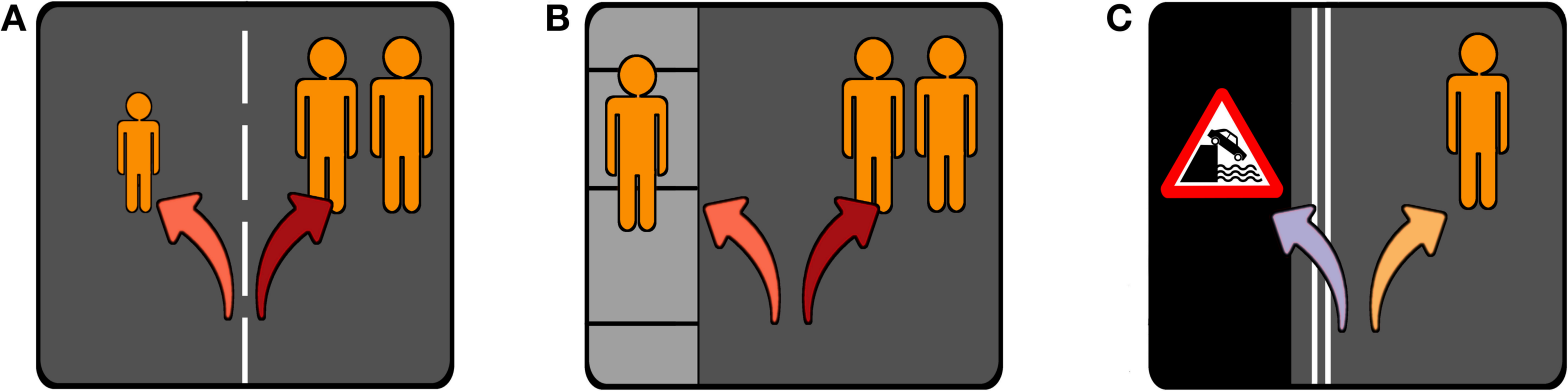
Pictorial representations of the three scenarios in Study 1. The relative numbers of orange figures in each scenario represent the ratios between the two groups at risk (assuming a single car occupant). The arrows indicate possible car actions and are colored corresponding to the graphs in Figure 2. **(A)** Children vs. adults scenario; **(B)** Sidewalk vs. road scenario; **(C)** Car occupants vs. pedestrians scenario.

We chose these specific types of scenarios as they allow us to contribute to related findings and discussions in recent literature. The influence of potential victims' ages has been investigated by Sütfeld et al. (2017), Awad et al. (2018), and Faulhaber et al. (2018) (further elaborated by Bergmann et al., 2018). The potential protection afforded to pedestrians on a sidewalk has been studied in Faulhaber et al. (2018) (further elaborated by Bergmann et al., 2018). The issue of prioritizing car occupants or pedestrians has been theoretically discussed by Lin (2015) and Gogoll and Müller (2016), and implemented in a multitude of experiments including Bonnefon et al. (2016), Wintersberger et al. (2017), Awad et al. (2018), Faulhaber et al. (2018) (further elaborated by Bergmann et al., 2018), and Ju et al. (2019). The three scenarios are conceptually depicted in Figure 1 and details of the trials for each scenario are shown in Table 1.

**Table 1:** Outline of trials for Study 1.

**Scenario**	**Trial**	**Groups at risk**
Children vs.Adults	Smaller groups	1 child (+ viewpoint avatar^†^) vs. 2 adults (+ viewpoint avatar^†^)
	Larger groups	2 children (+ viewpoint avatar^†^) vs. 4 adults (+ viewpoint avatar^†^)
Sidewalk vs.Road	Smaller groups	1 adult on sidewalk vs. 2 adults on road
	Larger groups	2 adults on sidewalk vs. 4 adults on road
Car occupants vs.Pedestrians	Parked van	2 adult car occupants vs. 2 adults on road
	Cliff	2 adult car occupants vs. 2 adults on road

† *To avoid the artificiality of presenting the scenarios from the perspective of a child, additional adult avatars were added to both groups in the children vs. adults scenario, from which the pedestrian perspectives were presented*.

As described, the numbers of lives at risk were unequal in the first two scenarios. There were twice as many pedestrians on the road compared to the sidewalk, and twice as many adults as children. These particular ratios were chosen based on the results from the study reported by Faulhaber et al. (2018), which were further elaborated by Bergmann et al. (2018). The number of car occupants and pedestrians at risk were equal in the car occupants vs. pedestrians scenario. This ratio was anticipated to best elicit differences between the car occupant and pedestrian perspectives, as, barring any intrinsic bias toward pedestrians or car occupants, both should be equally valued.

#### 2.1.3. Design

We employed a 4 (perspective) × 2 (motorist-type) between-participants factorial design. The two levels of motorist-type were self-driving car and human driver. The four levels of perspective were passenger, observer, pedestrian in the smaller group and pedestrian in the larger group. We used a between-participant design to prevent experimental confounds such as recognition of the trials and attempts to be self-consistent. As decisions made during previous trials could be easily recalled, we considered that a within-participant design would not have allowed us to distinguish whether participants were influenced more by the experimental manipulations or by their previous responses. Thus, variables were manipulated in such a way that each participant saw all trials from the same perspective and involving the same motorist-type. To control for gender effects such as those described by Skulmowski et al. (2014), the genders of all human avatars in the virtual environment were matched to each participant.

#### 2.1.4. Procedure

Participants were assigned via permuted block randomization to one of the eight conditions corresponding to the combinations of perspective and motorist-type (e.g., observer and human driver; car occupant and self-driving car). Participants of the smaller and larger pedestrian groups shared the same car occupants vs. pedestrians trials as there was only one pedestrian group involved in those scenarios. Participants completed a practice trial and a control trial before the experimental trials. The six experimental trials as well as animations within each trial were shown in random order; trials were separated by distraction tasks. After viewing a pair of animations, participants could replay the pair as many times as they wanted. Participants were then asked to choose which of the two actions of the motorist they considered to be more acceptable by selecting the corresponding outcome image. In accordance with Mandel and Vartanian (2007), after making each judgement, participants indicated how confident they were in it on a scale from 0 (not confident at all) to 100 (very confident). Decision confidence in moral dilemmas has also been previously investigated by Parkinson et al. (2011), Royzman et al. (2014), and Lee et al. (2018), as it gives further information than merely the binary choice. Specifically, the confidence ratings provide information on how conflicted participants were about the corresponding judgements. High scores on confidence indicate more robust judgements than lower scores. Thus, the proportions of judgements and the corresponding confidence levels should be considered in parallel.

After the experiment ended, participants completed a short questionnaire on demographics, driving experience, prior knowledge of self-driving cars and their attitudes toward them. Furthermore, as a manipulation check, participants reported which party in the situation they identified most with while watching the animations by responding to the question “while watching the animations, which party did you identify most strongly with?”. The options were the pedestrians, the car occupants or the observer. Finally, they were asked whether the motorist was a human driver or a self-driving car. Those participants who failed the control task or were not able to recollect the correct motorist-type in the self-driving car condition were excluded.

#### 2.1.5. Statistical Analysis

Statistical analyses were conducted in R (R Core Team, 2018) using *lme4* (Bates et al., 2015) for model fitting. Significance testing was performed using parametric bootstrapping with *afex* (Singmann et al., 2018) and *emmeans* (Lenth, 2018) was used for follow-up multiple comparisons on the estimated marginal means (*EMM*s).

Two models were computed for each of the three scenarios: one for the prediction of judgements (which of the two actions was considered more acceptable); the other for participants' self-reported confidence in their own judgements. Judgements, based on perspective and motorist-type, were modeled by logit mixed models. As there were two trials per participant for each dilemma, random by-participant intercepts were included in all models. This corresponds to the maximal random effects structure as described by Barr (2013) and Barr et al. (2013). Significance testing using Type-III sums of squares was performed by parametric bootstrapping with 1,000 simulations. Confidence, based on judgement, perspective, motorist, and trial was modeled by linear mixed models. Significance testing using Type-III sums of squares was performed using Kenward-Roger test. Along with trial (smaller groups/larger groups in the first two scenarios, parked van/cliff in the third scenario), the following covariates were included: gender, age, positive opinion of self-driving cars, visual acuity, education level, and driving experience. Models without covariates are reported in the Supplementary Material, but did not result in different conclusions. Results for the three scenarios are reported separately.

### 2.2. Results

#### 2.2.1. Manipulation Check

To determine whether varying the visual perspective affected which party participants self-identified with, we performed a chi-squared test of independence, comparing participants' self-identification with the perspective from which they experienced the situations (Table S2). The majority of participants identified most strongly with the perspective from which they experienced the scenarios χ^2^(24, *N* = 184) = 114.11, *p* < 0.0001. Follow up Bonferroni-adjusted comparisons showed all three perspective groups had significantly different patterns of responses from each other (all *p* < 0.0001) (Table S3). Thus, the manipulation check indicates that in most cases participants identified with the intended perspective.

#### 2.2.2. Children vs. Adults

**Figure 2:**
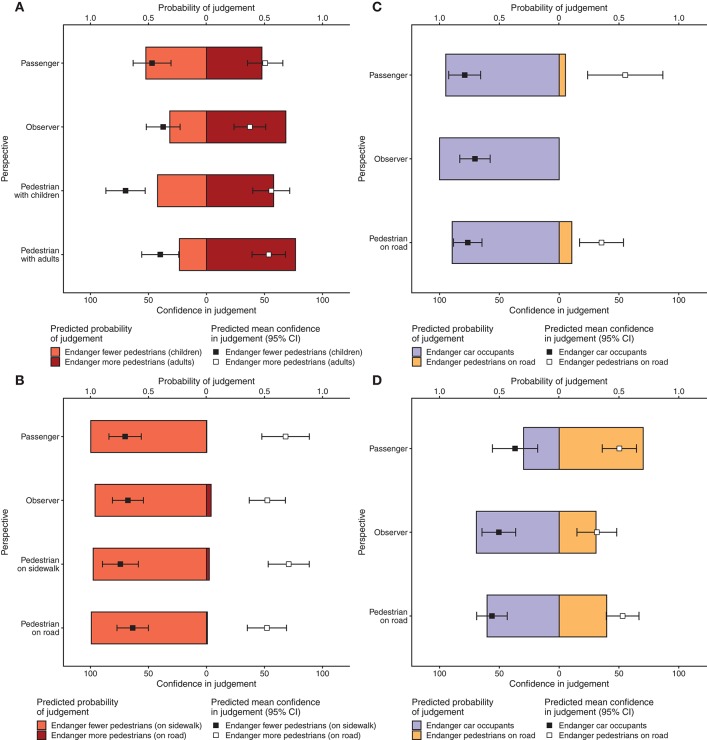
Model predictions for judgements and confidence (Study 1). Colored bars indicate the predicted probability of making particular judgements (indicated on the top x-axis) and are colored corresponding to the actions shown in Figure 1. Black and white squares with error bars indicate predicted mean self-reported confidence (95% CI) in the judgements made on a 0–100 scale (indicated on the bottom x-axis). As there were no significant effects of motorist-type, predictions are only separated by perspective. **(A)** Children vs. adults scenario; **(B)** Sidewalk vs. road scenario; **(C)** Car occupants vs. pedestrians scenario (parked van trial)—note there were no observers who preferred endangering pedestrians, so the confidence in that case could not be estimated; **(D)** Car occupants vs. pedestrians scenario (cliff trial).

Next, we investigated the influence of perspective and motorist-type on judgements on the children vs. adults scenario. According to model predictions, endangering the larger group, which consisted of only adult pedestrians, was considered more acceptable than endangering the smaller group, which consisted of adults and children (probability = 0.71). Figure 2A depicts the predicted probability of judgements and levels of confidence separated by perspective and motorist-type based on the statistical model. There were no significant effects of perspective or motorist-type on judgements (Table 2). The predicted mean self-confidence in judgements (on a 0–100 scale) was 49.92, however it varied considerably between conditions. There was a significant main effect of perspective (*p* = 0.0017) moderated by judgement (*p* = 0.0222) on self-reported confidence in judgements (Table 3). Within those who chose endangering the larger group (of only adults) as more acceptable, participants in the observer perspective had significantly lower confidence in their choices (*EMM* = 35.86) than either the pedestrian with children (*EMM* = 58.57) or the pedestrian with adults (*EMM* = 55.62) perspectives, *p* = 0.0178, *p* = 0.0358, respectively. Within those who chose endangering children as more acceptable, participants in the pedestrian with children perspective had significantly greater confidence (*EMM* = 71.87) than either the observer (*EMM* = 36.13), the passenger (*EMM* = 41.92), or the pedestrian with adults (*EMM* = 42.34), *p* = 0.0003, *p* = 0.0161, *p* = 0.0045, respectively (Tables S4, S5). Thus, observers had among the lowest confidence regardless of judgement.

**Table 2:** Predictors of judgements based on separate logit mixed models for each scenario (Study 1). *p*-values are calculated by parametric bootstrapping with 1,000 samples.

	**χ^2^**	**df**	***p***
**Children vs. adults scenario**			
Perspective	2.92	3	0.5205
Motorist-type	3.57	1	0.0991
Trial	1.22	1	0.2475
Perspective × motorist-type	1.60	3	0.7293
Gender	0.58	1	0.4635
Age	0.38	1	0.5972
Positive opinion of self-driving cars	11.33	4	0.0639
Education	4.47	2	0.1968
Driving experience	5.60	3	0.2070
Visual acuity	6.05	2	0.0859
**Sidewalk vs. road scenario**			
Perspective	6.94	3	0.0986
Motorist-type	3.70	1	0.0744
Trial	5.11	1	0.0543
Perspective × motorist-type	5.50	3	0.1698
Gender	5.15	1	0.0253^*^
Age	0.65	1	0.4200
Positive opinion of self-driving cars	7.51	4	0.0866
Education	4.37	2	0.1512
Driving experience	6.06	3	0.1040
Visual acuity	3.81	2	0.1170
**Car occupants vs. pedestrians scenario**			
Perspective	5.12	2	0.1399
Motorist-type	3.45	1	0.0909
Trial	68.89	1	0.0010^**^
Perspective × motorist-type	3.43	2	0.2452
Perspective × trial	8.58	2	0.0170^*^
Motorist-type × trial	2.64	1	0.1515
Perspective × motorist-type × trial	6.48	2	0.0630
Gender	0.05	1	0.8417
Age	0.62	1	0.4754
Positive opinion of self-driving cars	5.40	4	0.3083
Education	1.98	2	0.4230
Driving experience	3.28	3	0.4210
Visual acuity	5.68	2	0.0960

* *p < 0.05*,

** *p < 0.01*.

**Table 3:** Predictors of self-reported confidence based on separate linear mixed models for each scenario (Study 1). *p*-values are calculated by Kenward-Roger test.

	**Num df**	**Den df**	***F***	***p***
**Children vs. adults scenario**				
Perspective	3	169	5.27	0.0017^**^
Motorist-type	1	169	1.50	0.2230
Decision	1	325	0.09	0.7600
Trial	1	180	0.24	0.6275
Perspective × motorist-type	3	170	0.55	0.6509
Perspective × judgement	3	322	3.25	0.0222^*^
Motorist-type × decision	1	329	1.55	0.2139
Perspective × motorist-type × judgement	3	320	2.25	0.0823
Gender	1	164	0.04	0.8500
Age	1	159	1.68	0.1970
Positive opinion of self-driving cars	4	161	0.52	0.7180
Education	2	164	0.13	0.8825
Driving experience	3	161	0.28	0.8373
Visual acuity	2	163	0.63	0.5337
**Sidewalk vs. road scenario**				
Perspective	3	191	2.30	0.0791
Motorist-type	1	191	0.03	0.8542
Judgement	1	338	4.57	0.0332^*^
Trial	1	180	1.73	0.1900
Perspective × motorist-type	3	190	1.92	0.1279
Perspective × judgement	3	332	0.78	0.5044
Motorist-type × judgement	1	338	2.47	0.1170
Perspective × motorist-type × judgement	3	332	2.12	0.0979
Gender	1	164	2.95	0.0875
Age	1	160	0.02	0.8910
Positive opinion of self-driving cars	4	161	1.10	0.3607
Education	2	161	0.23	0.7982
Driving experience	3	161	0.50	0.6810
Visual acuity	2	160	2.86	0.0603
**Car occupants vs. pedestrians scenario**				
Perspective	2	250	1.07	0.3457
Motorist-type	1	284	0.20	0.6534
Judgement	1	326	13.77	0.0002^***^
Trial	1	248	7.93	0.0052^**^
Perspective × motorist-type	2	232	0.19	0.8263
Perspective × judgement	2	327	1.69	0.1866
Motorist-type × judgement	1	322	0.68	0.4118
Perspective × trial	2	242	2.49	0.0852
Motorist-type × trial	1	258	0.00	0.9652
Judgement × trial	1	298	10.81	0.0011^**^
Perspective × motorist-type × judgement	2	321	0.16	0.8508
Perspective × motorist-type × trial	2	236	0.18	0.8339
Perspective × judgement × trial	2	287	0.49	0.6112
Motorist-type × judgement × trial	1	301	0.07	0.7974
Perspective × motorist-type × judg. × trial	1	303	0.17	0.6827
Gender	1	164	0.54	0.4627
Age	1	164	0.51	0.4752
Positive opinion of self-driving cars	4	164	1.21	0.3074
Education	2	161	4.06	0.0191^*^
Driving experience	3	165	0.53	0.6639
Visual acuity	2	166	0.17	0.8457

* *p < 0.05*,

** *p < 0.01*,

*** *p < 0.001*.

#### 2.2.3. Sidewalk vs. Road

In the second scenario, we tested small groups of pedestrians on the sidewalk against larger groups of pedestrians on the road. Overall, endangering the smaller group was considered more acceptable than endangering the larger group (probability = 0.84). Thus, participants overwhelmingly considered that endangering fewer pedestrians was more acceptable, despite those pedestrians being situated on a sidewalk. Mean confidence (on a 0–100 scale) was 62.44 and, thus, considerably greater than in the children vs. adults scenario. Figure 2B depicts the predicted probability of judgements and levels of confidence separated by perspective and motorist-type based on the model. There were no significant effects of perspective or motorist type on judgements (Table 2). However, there was a significant effect of gender, such that females (probability = 0.004) were less likely to consider endangering the larger group of pedestrians (on the road) as more acceptable than males (probability = 0.034). Self-reported confidence depended on judgement (Table 3), such that choosing endangering pedestrians on the sidewalk as more acceptable was associated with greater confidence (*EMM* = 68.88) than choosing endangering pedestrians on the road (*EMM* = 60.93), *p* = 0.0332 (Tables S6, S7). Thus, the observed differences in confidence matches the bias in judgement in the sidewalk vs. road scenario.

#### 2.2.4. Car Occupants vs. Pedestrians

Finally, we investigated a scenario in which endangering car occupants was contrasted with endangering pedestrians. As the two trial types for this scenario were conceptually different, an interaction with trial type was included in the model.

For the parked van trial, the vast majority preferred to endanger the car occupants (probability = 0.99). In the cliff trial however, this was much less likely (probability = 0.53). Mean confidence was also different: 67.08 for the parked van trial and 43.62 for the cliff trial. Figure 2C depicts the predicted probability of judgements and levels of confidence separated by perspective and motorist-type for the parked van trial and Figure 2D depicts the same for the cliff trial.

There was a significant main effect of trial-type. Participants were more likely to consider endangering the car occupants as more acceptable in the van trial than the cliff trial, *p* = 0.0010. As falling off a cliff is more likely to result in injury or death than colliding with a parked van, the judgements by participants appear to take into account the degree of potential harm.

Furthermore, there was a significant trial-type × perspective interaction. In the cliff trial, passengers were significantly less likely than either observers (odds-ratio = 5.303, *p* = 0.0047) or pedestrians (odds-ratio = 3.584, *p* = 0.0118) to consider endangering the car occupants (including themselves) as more acceptable. This indicates a self-preservation effect.

Statistical analysis of self-reported confidence was performed only for pedestrians and car occupant perspectives as there were no responses preferring to endanger pedestrians in the observer perspective. There were main effects of trial (*p* = 0.0052) and judgement (*p* = 0.0002), moderated by a trial × judgement interaction (*p* = 0.0011), on self-reported confidence. Confidence when preferring to endanger car occupants was lower in the cliff trial (*EMM* = 47.8) than the parked van trial (*EMM* = 75.2), *p* < 0.0001. This was not the case for preferring to endanger pedestrians (*EMMs* = 50.4 and 55.2, respectively, *p* = 0.7582) (Table S11). Note that there were no observers who preferred endangering pedestrians in the parked van trial, so the confidence could not be estimated and the follow up comparisons for endangering pedestrians only considered the responses of the other perspectives.

### 2.3. Study 1 Discussion

For the three scenarios, patterns of judgements aligned with actions taken in similar dilemma studies reported by Faulhaber et al. (2018) (further elaborated by Bergmann et al., 2018) and Sütfeld et al. (2017): participants generally preferred motorists to risk the lives of adult pedestrians rather than child pedestrians, despite endangering more lives by doing so; it was highly acceptable for a motorist to swerve onto a sidewalk in order to endanger fewer pedestrians; and there was a tendency to protect pedestrians over car occupants. However, it seems that the perceived danger to the car occupants plays a role; participants were less likely to accept a car veering toward a cliff edge, than a car veering toward a parked van.

Only in the cliff trial of the car occupants vs. pedestrians scenario did we observe a main effect of perspective on judgements. There was disagreement between the car occupant and pedestrian perspectives. Car occupants preferred the car to remain on course and endanger the pedestrians, rather than veering toward a cliff edge, while pedestrians preferred the opposite. Interestingly, observers appear to agree with the pedestrians in this case. This corresponds to a self-preservation effect for both car occupants and pedestrians. However, it is important to notice that this effect only arose when the situation clearly pitted the lives of car occupants against the lives of pedestrians. It was not prevalent between pedestrians, nor in the parked van trial (which may have been considered as less dangerous for the car occupants).

The collection of self-reported confidence allowed for a more fine-grained analysis by enabling effects that were not prevalent in the primary forced-choice response data to be investigated. Specifically, there was an effect of perspective in the children vs. adults scenario: observers were among the lowest in confidence, regardless of judgement, despite there being no significant difference in judgements themselves. This is noteworthy as the uninvolved observer is often considered as an “objective” viewpoint (Coeckelbergh, 2016). One might then expect the observer perspective to be associated with high confidence, but this is not apparent here.

## 3. Study 2—Moral Judgements on Simplified Animations

Our second study builds on the first investigating the influence of perspective and motorist with the addition of investigating the influence of the number of lives at risk and the presence of a sidewalk. We used an online deployment platform and presented the scenarios in the form of simplified animations. Rather than offering an immersive experience, the goal of using simplified animations was to illustrate the scenarios while prompting participants to evaluate them from a particular perspective. We consider the use of animations to be a natural extension of the combination of simplified images and textual vignettes, as used in previous studies (Bonnefon et al., 2016; Li et al., 2016; Awad et al., 2018). As such a combination has been shown by Sachdeva et al. (2015) to sufficiently manipulate perspective in moral dilemmas, simplified animations should similarly prompt participants to consider situations from the presented perspective. Nevertheless, a manipulation check was included in the analysis to confirm that such an effect occurred.

We tested whether increasing the number of lives at risk by staying on course increases the acceptability of swerving to endanger a single life. Further, we tested whether swerving onto a sidewalk would be less acceptable than swerving onto another road. We hypothesized that perspective would influence judgements, such that participants would be less likely to consider endangering their own avatars as the more acceptable action.

### 3.1. Materials and Methods

#### 3.1.1. Participants

Three hundred and sixty-eight people (176 male, 191 female, 1 other) voluntarily participated in this online animation-based experiment. Participants indicated their age groups, the median of which was 18–29 years old. Participants were recruited through social media, university mailing lists and word of mouth. Twenty-four different countries were represented, with major participation from Germany, Armenia, Australia, and Russia. The study was approved by the ethics review board at Osnabrück University, Germany. Descriptive statistics of the participants are given in Table S12.

#### 3.1.2. Materials

**Figure 3:**
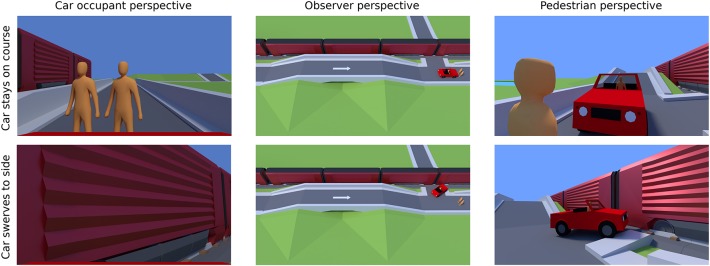
Final frames from animations for the pedestrians vs. car occupant scenario (Study 2). The car either stays on course, endangering two pedestrians (top row), or swerves into a freight train, endangering the car occupant (bottom row). Different perspectives are shown: car occupant perspective (left column), observer perspective (middle column), pedestrian perspective (right column). Images depict 2v1 lives-at-risk (2 pedestrians vs. 1 car occupant). The animations used graphical models based on those by Jim van Hazendonk (https://racoon.media/) and Clint Bellanger (http://clintbellanger.net/).

The stimuli consisted of animations of five seconds in length made with Blender (Blender Online Community, 2018). Each animation depicted a car traveling over a hill. Immediately after the hill, the car encountered a dilemma situation. It could either stay on course and risk the lives of pedestrians on the road or swerve to the side. Depending on the scenario, swerving would direct the car either into a single pedestrian (on a road or a sidewalk) or the side of a passing freight train. The animations ended shortly before impact to avoid unnecessary distress for participants. To manipulate the perspective, each animation depicted a scenario from either a bird's-eye view; a first-person perspective of a pedestrian; or a first-person perspective of the car occupant (Figure 3).

#### 3.1.3. Design

Two scenarios were investigated in this study (pedestrian vs. pedestrian; car occupants vs. pedestrians). While the two associated designs differed in important ways, the general framework was the same. Four different lives-at-risk situations were investigated; swerving always endangered a single life, but staying on course endangered from 1 to 4 lives, depending on the trial.

For the pedestrians vs. single pedestrian scenario we employed a 2 (motorist-type) × 4 (perspective) × 2 (road-type) × 4 (lives-at-risk) mixed factorial design. There were two levels of motorist-type (self-driving car, human driver), and four of perspective (car occupant, pedestrian-straight-ahead, pedestrian-on-the-side, observer). All participants saw the two levels of road-type (split-road, road-with-sidewalk) and lives-at-risk (1 vs. 1, 2 vs. 1, 3 vs. 1, and 4 vs. 1). Motorist-type and perspective were manipulated between-participants, while road-type and lives-at-risk were manipulated within-participants. Thus, each participant witnessed all pedestrians vs. single pedestrian scenario from a single perspective involving a single motorist-type.

For the pedestrians vs. car occupant scenario we employed a 2 (motorist-type) × 3 (perspective) × 4 (lives-at-risk) mixed factorial design. Motorist-type had two levels (self-driving, human-driven) and perspective had three levels (car occupant, pedestrian straight ahead, observer). All participants saw all four levels of lives-at-risk (1 vs. 1, 2 vs. 1, 3 vs. 1, and 4 vs. 1). Motorist-type and perspective were manipulated between-participants, while lives-at-risk was manipulated within-participants. Thus, each participant witnessed all occupant vs. pedestrian dilemmas from a single perspective involving a single motorist-type.

#### 3.1.4. Procedure

Participants were given a link to an animation-based online survey, created and hosted on LabVanced, an online platform for social science experiments (Finger et al., 2017). Upon starting the study, participants were randomly allocated into one of the eight conditions described above, corresponding to the combinations of motorist-type and perspective in the larger design. Participants in observer and car occupant perspectives were presented both scenarios, as described above. However, the participants allocated to the pedestrian on-the-side perspective did not view the pedestrians vs. car occupant scenario, as there was no corresponding viewpoint in these animations. A single trial consisted of a pair of animations depicting the same situation. One animation showed the car staying on course, the other showed it swerving to the side. The order of the two animations was counterbalanced across trials. After viewing the pair of animations, images of the final frames of each animation were presented side-by-side. Participants were asked to choose which of the two actions was more acceptable by clicking on the corresponding image. Throughout the trials, a textual notice reminded participants about both the perspective from which they are viewing the scenarios and the type of motorist depicted.

All experimental trials were completed in random order. The experiment always began with a control trial; participants who failed it were excluded. After the experimental block, participants completed a short questionnaire on demographics, driving experience, prior knowledge of self-driving cars, and opinion toward them. Furthermore, participants were asked whether they identified more with the pedestrians or the car occupant while watching the animations with the question: “while watching the animations, which party did you most strongly identify with?” The options were: the car, the pedestrians.

#### 3.1.5. Statistical Analysis

As with the first study, statistical analyses were conducted in R (R Core Team, 2018) using *lme4* (Bates et al., 2015) for model fitting. Significance testing was performed using likelihood ratio tests with *afex* (Singmann et al., 2018) and *emmeans* (Lenth, 2018) was used for follow-up multiple comparisons on the estimated marginal means (*EMM*s).

Following the study design, the two scenarios were analyzed individually. For both, we modeled the likelihood of choosing swerving to the side as more acceptable than staying on course based on lives-at-risk, road-type, perspective and motorist-type, using generalized linear mixed models with logit link functions. To control for individual differences, we implemented maximal random-effects structures as suggested by Barr (2013) and Barr et al. (2013). In the pedestrian vs. pedestrian dilemmas, due to convergence issues, the maximal random effects structure was replaced with a sub-maximal structure, without the random slope for lives-at-risk. The following covariates were included in all models: gender, age, knowledge of self-driving cars, and opinion of self-driving cars.

### 3.2. Results

Similar to Study 1, we first performed a manipulation check to determine if the perspective from which participants viewed the scenarios affected with which party they identified most strongly. The omnibus goodness-of-fit test was significant, χ^2^(24, *N* = 350) = 60.66, *p* < 0.0001. The majority of participants in the pedestrian or car occupant perspectives identified most strongly with the corresponding perspective. Approximately equal numbers of participants in the observer perspective identified with car occupants and pedestrians (Tables S13, S14). Thus, the manipulation check indicates that in most cases participants identify with the allocated perspective and the observer perspective was not biased.

Next, we investigated the effects of the perspective, motorist-type, road-type and lives-at-risk on judgements on the pedestrian vs. pedestrian dilemma (Table 4). There was a significant main effect of lives-at-risk (*p* < 0.0001). With increasing imbalance of the number of pedestrians endangered, the probability of swerving changed steeply from close to 0.0 to nearly 1.0. Further, we observed a significant main effect of road-type (*p =* 0.0002). Participants tended to perceive swerving as more acceptable when swerving onto another road (probability = 0.88) than onto a sidewalk (probability = 0.76), odds-ratio = 2.50 (Table S16).

**Table 4:** Predictors of judgements based on separate logit mixed models for each scenario (Study 2). *p*-values are calculated via likelihood ratio tests.

	**df**	**χ^2^**	**χ^2^ df**	***p***
**Pedestrians vs. single pedestrian scenario**				
Lives-at-risk	46	899.92	3	<0.0001^***^
Perspective	46	2.99	3	0.3928
Motorist-type	48	2.19	1	0.1389
Road-type	48	9.87	1	0.0017^**^
Lives-at-risk × perspective	40	70.19	9	<0.0001^***^
Lives-at-risk × motorist-type	46	1.72	3	0.6316
Perspective × motorist-type	46	0.96	3	0.8108
Lives-at-risk × road-type	46	2.97	3	0.3956
Motorist-type × road-type	48	0.98	1	0.3214
Lives-at-risk × perspective × motorist-type	40	20.47	9	0.0152^*^
Lives-at-risk × motorist-type × road-type	46	0.84	3	0.8409
First animation	48	0.01	1	0.9305
Positive opinion of self-driving cars	45	12.92	4	0.0117^*^
Knowledge of self-driving cars	48	1.29	1	0.2566
**Pedestrians vs. car occupant scenario**				
Lives-at-risk	28	123.35	3	<0.0001^***^
Perspective	29	1.95	2	0.3767
Motorist-type	30	0.94	1	0.3319
Lives-at-risk × perspective	25	7.13	6	0.3086
Lives-at-risk × motorist-type	28	6.93	3	0.0742
Perspective × motorist-type	29	2.36	2	0.3079
Lives-at-risk × perspective × motorist-type	25	14.07	6	0.0288^*^
First animation	30	0.01	1	0.9190
Positive opinion of self-driving cars	27	10.20	4	0.0371^*^
Knowledge of self-driving cars	30	5.71	1	0.0168^*^

* *p < 0.05*,

** *p < 0.01*,

*** *p < 0.001*.

**Figure 4:**
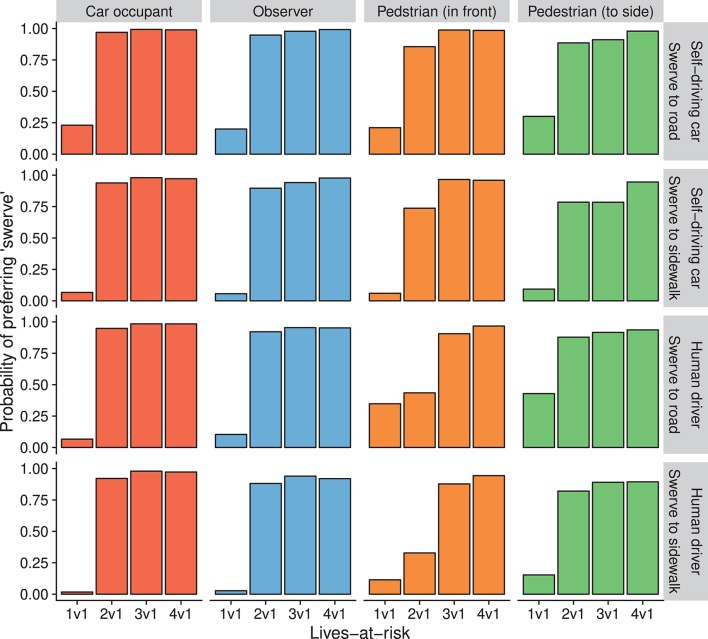
Model predictions for judgements on the pedestrians vs. single pedestrian scenario (Study 2). Height of bars indicate the probability of choosing “swerve” (endanger a single pedestrian to the side) as more acceptable. Different perspectives are separated in columns, combinations of motorist-type, and road-type are separated in rows.

Generally, increases in lives-at-risk were positively associated with the probability of preferring to swerve (the more lives at risk by staying, the higher the probability of preferring to swerve). However, the nuances of this relationship depended on perspective and motorist-type and their interaction (Table S17). Lives-at-risk interacted with perspective (*p* < 0.0001) and we observed a three-way interaction of lives-at-risk × perspective × motorist-type (*p* = 0.0152) (Figure 4). Specifically, comparing the case of 2v1 lives-at-risk, the probability of swerving was higher for car occupant and observer perspectives than for pedestrian perspectives. Furthermore, there was a difference in the case of 2v1 lives-at-risk from the pedestrian-straight-ahead perspective between human driver and self-driving car. Follow up comparisons of the lives-at-risk × perspective × motorist-type interaction indicated that in all except one condition, acceptability of swerving was significantly higher at 2 vs. 1 compared to 1 vs. 1 lives-at-risk, all *p* < 0.0001 (Table S18). The exception to this was for participants who judged human drivers from the perspective of pedestrians-straight-ahead. In their case, this increase only occurred at 3 vs. 1 lives-at-risk (odds-ratio = 31.67, *p* < 0.0001). This indicates that perspective may affect how human drivers' actions are perceived, and at which point it is considered appropriate for them to intervene.

**Figure 5:**
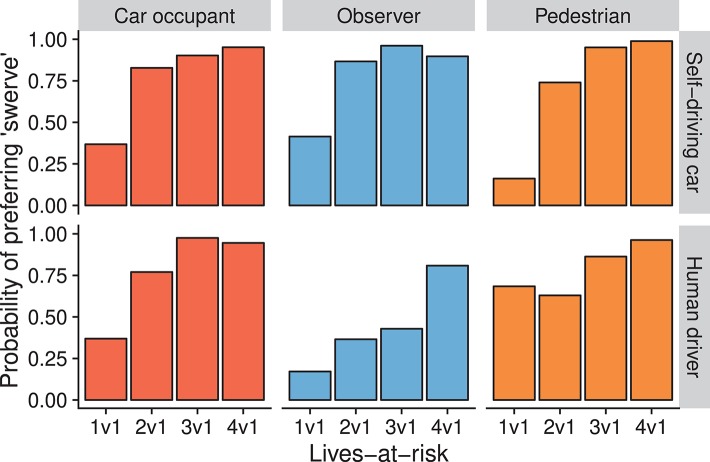
Model predictions for judgements on the pedestrians vs. car occupant scenario (Study 2). Height of bars indicate the probability of choosing “swerve” (endanger the car occupant) as more acceptable. Different perspectives are separated in columns, different motorist-types are separated in rows.

In the next scenario, car occupants were weighed against pedestrians. There was a significant main effect of lives-at-risk (*p* < 0.0001) and a significant lives-at-risk × perspective × motorist-type interaction (*p* = 0.0288) (Table 4). Preferring to swerve was generally positively associated with lives-at-risk. In all conditions, swerving was significantly more acceptable at 4 vs. 1 lives-at-risk compared to 1 vs. 1 lives-at-risk (all *p* < 0.05). For judgements on self-driving cars this increase occurred between 1 vs. 1 and 2 vs. 1 lives-at-risk, while for judgements on human drivers, this point depended on perspective. For those in the car occupant perspective, there was no significant difference between 1 vs. 1 and 2 vs. 1 lives-at-risk (*p* = 0.0604), but there was a significant difference between 1 vs. 1 and 3 vs. 1 conditions (odds-ratio = 68.02, *p* = 0.0001). For both observers and pedestrians, this occurred only after 4 vs. 1 lives-at-risk, odds-ratios = 20.42 (*p* = 0.0011) and 11.97 (*p* = 0.0136), respectively. However, in the latter case, this was due to the already high acceptability of swerving at 1 vs. 1 lives-at-risk (probability = 0.68). These results are depicted in Figure 5. Thus, moral judgements were rather similar in the case of self-driving cars, and were dependent on perspective only in the case of human drivers.

### 3.3. Study 2 Discussion

In this study we observed that increasing the number of people in the direct path of a car led to higher acceptability of swerving to endanger a single life. Generally, when two or more pedestrians were in danger, the probability of preferring to swerve was substantially higher than when there was only a single pedestrian in danger. This is in line with previous studies, reporting a high sensitivity of participants to the number of lives at risk. Further, we observe that swerving onto a sidewalk was less acceptable than swerving onto a connecting road. However, this effect was overshadowed by the preference to minimize the number of lives endangered. Additionally, we observed other differences between judgements on human drivers and self-driving cars. When swerving would endanger a pedestrian, there was general agreement between perspectives for self-driving cars to minimize the number of lives endangered. However, for human drivers, this was not the case. Those in the perspective of pedestrians in the direct path of a car only accepted a human driver swerving when three or more pedestrians would be otherwise endangered. All other perspectives considered it more acceptable when there were two pedestrians in the direct path of a car (Figure 4). When swerving would endanger car occupants, there was general agreement between perspectives on what self-driving cars should do. It was more acceptable for self-driving cars to minimize harm while protecting their occupants when all else was equal. However, there was disagreement between perspectives about which action was more acceptable for human drivers to take. Those in the observer perspective only considered it more acceptable for drivers to endanger themselves when faced with four pedestrians on the road. Conversely, those in the pedestrian perspective already considered it more acceptable for drivers to swerve when there was a single pedestrian at risk (Figure 5). Similar to Study 1, this indicates a self-preservation effect for pedestrians, however only for judgements on human drivers.

## 4. General Discussion

In both studies, we found that judgements on self-driving cars do not seem to differ substantially from those on human drivers. In cases where there is a discrepancy, it seems to be due to a stronger preference for self-driving cars to minimize harm. Based on this result, it seems that people generally expect self-driving cars to follow the same traffic regulations as human drivers. The experiments revealed that differences between perspectives occur in situations where lives of car occupants are weighed against those of pedestrians. Results from Study 1 show that perspective seems to affect the acceptability of a car driving off a cliff: passengers are less likely to prefer swerving off a cliff than observers or pedestrians. Study 2 indicates disagreement between perspectives when considering at which point human drivers should intervene and endanger their own lives for the greater good. Additionally, perspective seems to affect confidence: people who observe a collision from a detached point of view seem to be less confident in their judgements. Although there are many commonalities in the judgements from different perspectives, the identified discrepancies should be taken into consideration in further research.

Results from our studies on moral judgement generally align with those from previous studies of moral action, in which participants were in the roles of drivers in similar dilemma scenarios (Sütfeld et al., 2017; Faulhaber et al., 2018). This indicates that the discrepancy between moral action and moral judgement (shown by e.g., Francis et al., 2016) may not be extremely pronounced in driving-related dilemmas presented in virtual environments. Thus, previous studies on the topic should be considered equally relevant irrespective of whether they focused on moral judgement or action.

One of the more controversial aspects of introducing self-driving cars may concern the endangering of pedestrians on sidewalks. According to our results, pedestrians on a sidewalk seem to be offered more protection than pedestrians on the road when the numbers of lives at risk are equal (Figure 4). However, this protection is overshadowed by the preference to endanger fewer lives (Figure 2B). This opposes prominent ethical guidelines such as those issued by the ethics commission of the German Federal Ministry of Transport and Digital Infrastructure (2017), which states that non-involved parties (e.g., pedestrians on a sidewalk) should not be endangered. Similar divergence occurs when a dilemma involves clearly risking the lives of car occupants or children, as there is no general agreement between people's judgements on what is considered more acceptable. However, the guidelines state that personal features, such as age, should not be taken into consideration in unavoidable accident situations. While ethical guidelines are important to consider, another aspect to consider is legality. In research by Awad et al. (2018) and Li et al. (2019), the legal liability of different parties involved in a situation (for example whether pedestrians were crossing legally or not) was shown to affect judgements. However these studies did not consider the interplay between the type of motorist, perspective and legality, something that future research should aim to elucidate.

Our studies aimed to expand understanding of moral psychology in the context of artificial intelligence. This research assists in determining criteria that self-driving car decision making needs to meet in order to be commonly accepted. However, we want to stress that responses to simplified dilemma situations should not be the basis for legal or ethical regulations. Furthermore, in agreement with Keeling (2017) and Nyholm (2018a), we believe empirical research alone cannot answer the ethical question of how self-driving cars should be programmed to behave. Nevertheless, we believe the results provide insights into the public's preferences regarding the decision making of self-driving cars and potential conflicts that may arise. The results from our studies point to specific questions warranting further investigation and attention in the debate surrounding the introduction of self-driving cars. In particular, these relate to the lack of agreement regarding specific dilemmas, apparent discrepancies between public opinion and ethical guidelines, the effects of perspective, the identified self-preservation effect and the albeit slight differences between judgements on self-driving cars and human drivers. These findings all highlight issues with creating decision making algorithms that attempt to simultaneously consider intuitions, ethical guidelines, and legal regulations.

## Data Availability Statement

The datasets generated for the studies are included in the Supplementary Material.

## Ethics Statement

The studies involving human participants were reviewed and approved by Ethics Commission of Osnabrück University. The participants provided their informed consent to participate in the studies.

## Author Contributions

PK, GP, and AS conceived of the initial research idea, gave feedback to the experimental designs, provided feedback and edited the manuscript. NK, FN, MP, and JZ participated in planning the research, collecting the data, designing the experiments, interpreting the results, and writing the manuscript. NK, FN, and MP participated in the creation of the materials. NK and MP analyzed the collected data.

### Conflict of Interest

The authors declare that the research was conducted in the absence of any commercial or financial relationships that could be construed as a potential conflict of interest.

## Supplementary Material

Data Sheet 1 Study 1 data.

Data Sheet 2 Study 2 data.

Data Sheet 3 Additional information for methods and results.
