# Supplementary material for: Moral Judgements on the Actions of Self-Driving Cars and Human Drivers in Dilemma Situations From Different Perspectives
Source: Front Psychol. 2019 Nov 1;10:2415. doi: 10.3389/fpsyg.2019.02415 (PMC6844247; doi:10.3389/fpsyg.2019.02415)
Supplement: Data Sheet 1 — Study 1 data. [file Data_Sheet_3.PDF]

Supplementary Material

1 EXTENDED PROCEDURE DESCRIPTIONS

1.1 Study 1

Participants were randomly assigned to one of the eight conditions corresponding to the combinations of perspective and motorist-type (e.g. observer & human driver; car occupant & self-driving car). Participants of the smaller and larger pedestrian groups shared the same pedestrians versus car occupants trials as there was only one pedestrian group involved in those scenarios.

Participants were asked to observe the environment carefully before pressing a button to begin each animation. There was a pause after the animation and sound stopped and participants could freely inspect the scene again before pressing a button to continue. The order of the animations within a trial was randomised for each participant. After viewing a pair of animations, participants could replay the pair in the same order as originally shown or continue to the response screen. There was no limit on the number of times the animations could be replayed before responding.

The response screen consisted of side-by-side images of the final frames of each animation. Participants were asked to choose which of the two actions of the motorist they considered to be more acceptable by selecting the corresponding outcome image. Depending on the motorist-type, the question either mentioned that the actions were taken by a person driving the car or a self-driving car. After making the judgement, participants indicated how confident they were with their choice on a scale from 0 (not confident at all) to 100 (very confident).

Participants completed a practice trial and a control trial before the experimental trials. The practice trial involved a scenario with a single pedestrian on one side of the road and a group of five pedestrians on the other side. This task allowed the participants to become accustomed to the immersion of VR and the controls.

The control trial consisted of a single pedestrian on one side and empty road on the other. The intention of the control trial was to check whether participants considered swerving to empty road more acceptable than endangering a single person. Participants were excluded if they considered swerving to the pedestrian as more acceptable than swerving to the empty side of the road, as this behaviour indicated a tendency to risk a life for no purpose or a misunderstanding of the task.

To separate each trial, distraction tasks were presented between them. These consisted of simple mathematical equations which participants had to indicate whether they were correct or incorrect. The distraction trial lasted 20 seconds regardless of the number of responses in that time. There was no visible countdown in order to avoid stress. The distraction trial ended once the participant responded to the final question displayed within the 20 seconds. No data from the distraction task was recorded or analysed.

After completing the practice and control trials, participants completed the six experimental trials in random order, separated by distraction tasks.

After the experiment ended, participants completed a short questionnaire on demographics, driving experience, prior knowledge of self-driving cars and their attitudes towards them. Furthermore, as a manipulation check, participants reported which party in the situation they identified most with while watching the animations: the pedestrians, the car occupants or the observer. Finally, they were asked

whether the motorist was a human driver or a self-driving car. Participants in the self-driving car condition that could not recollect their motorist-type were excluded, since this indicated they did not fully understand the instructions. We chose to not equally exclude the participants of the human driver condition as they were not briefed about different motorist-type conditions and only presented the term 'self-driving car' in the post-study questionnaire. On completion of the questionnaire, participants were debriefed. The entire procedure took approximately 25–30 minutes.

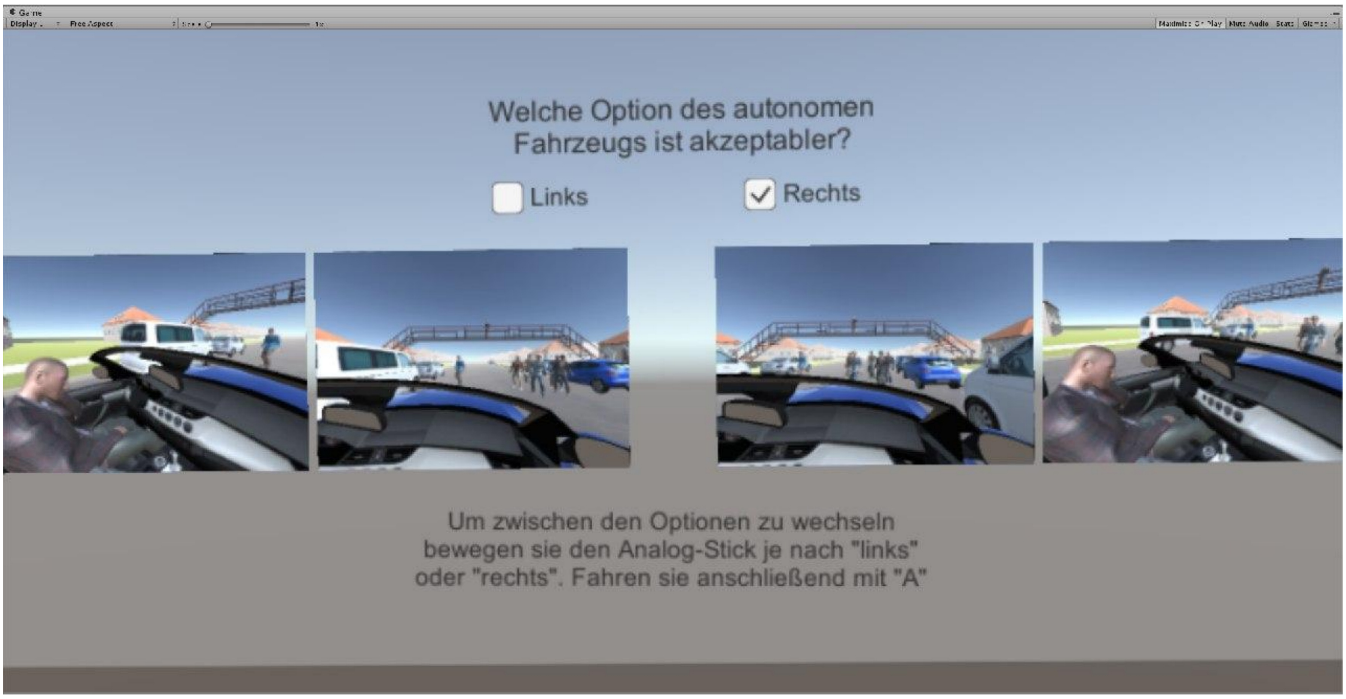

Figure S1: The screenshot depicts the decision screen presented to participants in the virtual reality study after have seen the animations. Two images of each choice were shown to show the scene from different angles. The scenario depicted here is a practice trial involving the choice between endangering a group of pedestrians or a single pedestrian. **(Left)** Self-driving car (with a relaxing second pedestrian) is veering towards one pedestrian; **(B)** Self-driving car is veering towards a group of pedestrians. Instructions translated from German: "Which option of the autonomous vehicle is more acceptable? (Left/Right) – To alternate between options, move the analog stick 'left' or 'right' respectively. Subsequently, continue with 'A'."

1.2 Study 2

Participants were given a link to an animation-based online survey, created and hosted on LabVanced, an online platform for social science experiments (Finger et al., 2017). Upon starting the study, participants were randomly allocated into one of the eight conditions mentioned before, corresponding to the combinations of motorist-type and perspective in the larger design. Participants were presented scenario

situations of both types, as described above. However, the participants allocated to the pedestrian on the side perspective did not view occupant-pedestrian scenarios, as there was no corresponding viewpoint in these animations.

A single trial consisted of a pair of animations depicting the same moral scenario situation. One animation showed the car staying on course, the other showed the car swerving to the side. The order of the two videos was counterbalanced across trials. After viewing the pair of animations, images of the final frames of each animation were presented side-by-side. Participants were asked to choose which of the two actions was more acceptable by clicking on the corresponding image. Depending on the motorist-type, the question either stated the actions were made by a person driving the car or a self-driving car. Below the question was a reminder of which perspective the animations were viewed from. Participants could click a button to replay the pair of animations in the same order as originally shown. There was no limit to the number of times the animations could be replayed before making a choice nor the amount of time participant needed to answer the up coming question.

The first trial was always a control task. The presented scenario involved a single pedestrian in the path of the car and a clear road to the side. Participants were excluded if they considered “stay” as more acceptable than “swerve”, as this indicated either a tendency to risk a life for no purpose or a misunderstanding of the task.

All participants completed eight trials of pedestrian vs. pedestrian scenarios, corresponding to the combinations of the levels of the road-type and lives-at-risk. An additional four trials were occupant vs. pedestrian scenarios, corresponding to the four different levels of lives-at-risk. However, participants assigned to the pedestrian on the side perspective only completed the trials relating to the pedestrian vs. pedestrian scenario. This was due to no corresponding viewpoint in the occupant vs. pedestrian scenario. Thus, participants completed a total of either eight or twelve trials. All experimental trials were completed in random order.

After the experimental block, participants completed a short questionnaire on demographics, driving experience, prior knowledge of self-driving cars and attitudes toward them. Furthermore, participants were asked whether they identified more with the pedestrians or the car occupant while watching the animations. The entire study procedure took approximately five minutes.

2 STUDY 1 SUPPLEMENTARY TABLES

Table S1: Descriptive statistics of the participants in Study 1.

motorist: human driver	observer	passenger	pedestrian (smaller group)	pedestrian (larger group)
gender (male:female)	12:11	12:11	12:11	12:11
age (M, SD)	23.57 (4.41)	21.87 (3.31)	23.35 (6.12)	23.04 (2.99)
driving experience				
no experience	6	3	3	4
1–5 years	6	15	16	9
5–10 years	10	4	2	9
≥10 years	1	1	2	1
education				
no higher education achieved	18	21	20	13
undergraduate education	3	2	3	10
post-graduate education	2	0	0	0
opinion of self-driving cars (M, SD)	3.52 (1.04)	3.48 (1.24)	3.48 (1.31)	3.48 (1.27)
visual acuity				
normal vision	17	13	15	9
corrected vision	5	6	6	12
uncorrected vision	1	4	2	2

motorist: self-driving car	observer	passenger	pedestrian (smaller group)	pedestrian (larger group)
gender (male:female)	12:11	12:11	12:11	12:11
age (M, SD)	23.30 (3.72)	21.70 (2.36)	22.43 (2.73)	24.26 (6.08)
driving experience				
no experience	4	4	2	3
1–5 years	9	14	13	11
5–10 years	8	5	7	6
≥10 years	2	0	1	3
education				
no higher education achieved	14	19	21	15
undergraduate education	6	4	2	6
post-graduate education	3	0	0	2
opinion of self-driving cars (M, SD)	3.30 (1.18)	3.43 (1.27)	3.52 (1.20)	3.3 (1.02)
visual acuity				
normal vision	13	13	15	10
corrected vision	9	7	7	10
uncorrected vision	1	3	1	3

Table S2: Contingency table for manipulation check (Study 1).

	identification		
perspective	observer	passenger	pedestrian
observer	31	10	5
passenger	8	35	3
pedestrian	33	3	56

Table S3: Follow up comparisons for manipulation check (Study 1).

	comparison	p	
1	observer vs. passenger	$< .01$	**
2	observer vs. pedestrian	$< .01$	**
3	passenger vs. pedestrian	$< .01$	**

Note: * $p < .05$, ** $p < .01$, *** $p < .001$.

Table S4: Estimated marginal means for self-reported confidence in judgements — children versus adults scenario (Study 1).

perspective	EMM	SE	df	lower 95% CI	upper 95% CI
judgement = endanger larger group (adults)					
observer	37.4348	6.9340	186.98	23.7558	51.1138
passenger	50.6119	7.7075	181.57	35.4042	65.8196
pedestrian (smaller group)	55.8615	8.0671	181.26	39.9441	71.7790
pedestrian (larger group)	53.7088	7.2938	181.68	39.3174	68.1002
judgement = endanger smaller group (children)					
observer	37.3025	7.3825	218.66	22.7525	51.8525
passenger	46.8662	8.2803	202.90	30.5397	63.1928
pedestrian (smaller group)	69.7271	8.5895	206.26	52.7926	86.6615
pedestrian (larger group)	39.8304	8.0855	226.03	23.8978	55.7630

Table S5: Follow up comparisons for self-reported confidence in children versus adults scenario (Study 1).

contrast	estimate	SE	df	t	p	
judgement = endanger larger group (adults)						
observer − passenger	−13.1771	7.2467	211.35	−1.818	.2676	
observer − pedestrian (smaller group)	−18.4267	7.0260	205.78	−2.623	.0459	*
observer − pedestrian (larger group)	−16.2740	6.8486	207.04	−2.376	.0849	
passenger − pedestrian (smaller group)	−5.2496	7.1644	214.91	−0.733	.8838	
passenger − pedestrian (larger group)	−3.0969	7.1190	207.64	−0.435	.9724	
pedestrian (smaller group) − pedestrian (larger group)	2.1528	6.9085	200.55	0.312	.9895	
judgement = endanger smaller group (children)						
observer − passenger	−9.5637	8.1973	260.82	−1.167	.6484	
observer − pedestrian (smaller group)	−32.4246	8.3792	276.43	−3.870	.0008	***
observer − pedestrian (larger group)	−2.5279	8.3234	286.57	−0.304	.9903	
passenger − pedestrian (smaller group)	−22.8608	8.2415	258.10	−2.774	.0301	*
passenger − pedestrian (larger group)	7.0358	8.5637	273.21	0.822	.8442	
pedestrian (smaller group) − pedestrian (larger group)	29.8966	8.7240	285.26	3.427	.0039	**

Note: * $p < .05$, ** $p < .01$, *** $p < .001$.

Table S6: Estimated marginal means for self-reported confidence in sidewalk versus road scenario (Study 1).

judgement	EMM	SE	df	lower 95% CI	upper 95% CI
endanger smaller group (sidewalk)	68.8838	5.8744	162.94	57.2840	80.4837
endanger larger group (road)	60.9278	6.6278	203.52	47.8599	73.9957

Table S7: Follow up comparisons for self-reported confidence in sidewalk versus road scenario (Study 1).

contrast	estimate	SE	df	t	p	
endanger smaller (sidewalk) − endanger larger (road)	7.9561	3.7212	337.76	2.138	.0332	*

Note: * $p < .05$, ** $p < .01$, *** $p < .001$.

Table S8: Estimated probability of preferring to endanger car occupants in car occupants versus pedestrians scenario (Study 1).

trial	P(“endanger car occupants”)	SE	df	lower 95% CI	upper 95% CI
parked van	0.9145	0.0403	Inf	0.7956	0.9671
cliff edge	0.5531	0.1078	Inf	0.3448	0.7442

Table S9: Follow up comparisons for estimated probability of preferring to endanger car occupants in car occupants versus pedestrians scenario (Study 1).

contrast	odds ratio	SE	df	z	p	
parked van / cliff edge	8.6435	2.9567	Inf	6.305	<.0001	***

Note: * $p < .05$, ** $p < .01$, *** $p < .001$.

Table S10: Estimated marginal means for self-reported confidence in car occupants versus pedestrians scenario (Study 1).

trial	EMM	SE	df	lower 95% CI	upper 95% CI
parked van	64.5887	5.5074	205.55	53.7304	75.4470
cliff edge	48.4315	5.2839	188.95	38.0085	58.8546

Table S11: Estimated Marginal Means for self-reported confidence separated by trial and judgement in car occupants versus pedestrians scenario (Study 1).

trial	EMM	SE	df	lower 95% CI	upper 95% CI
judgement = endanger pedestrians					
parked van	55.2472	15.9957	330.19	23.7809	86.7134
cliff	50.3588	7.2760	259.22	36.0312	64.6864
judgement = endanger car occupants					
parked van	75.2332	5.2659	192.35	64.8470	85.6195
cliff	47.8217	5.8862	237.91	36.2260	59.4175

Note that for the parked van trial, there were no observers preferring to endanger pedestrians, so the EMMs for that trial only consider the other perspectives.

3 STUDY 2 SUPPLEMENTARY TABLES

Table S12: Descriptive statistics of the groups in car occupants versus pedestrians scenario (Study 2).

age group	gender	knowledge of self-driving cars	driving experience	country
18–29: 142	female:151	no: 23	0: 52	Germany: 55
30–39: 43	male: 130	yes:259	5: 54	Armenia: 30
40–49: 18	NA’s: 1		6–10: 35	Australia: 25
50–59: 18			10+: 95	Russia: 10
60–69: 24			NA’s: 37	(Other): 19
70–79: 3				NA’s: 142
80–89: 1				

Table S13: Contingency table for manipulation check (Study 2).

	identification	
	car occupant	pedestrian
perspective		
car occupant	73	28
observer	53	42
pedestrian (in car’s path)	21	65
pedestrian (to side of car’s path)	23	63

Table S14: Follow up comparisons for manipulation check (Study 2).

comparison	p	
car vs. observer	.02	
car vs. pedestrian (in car's path)	<.01	**
car vs. pedestrian (to side of car's path)	<.01	**
observer vs. pedestrian (in car's path)	<.01	
observer vs. pedestrian (to side of car's path)	<.01	**
pedestrian (in car's path) vs. pedestrian (to side of car's path)	.86	

Note: * $p < .05$, ** $p < .01$, *** $p < .001$.

Table S15: Follow up comparisons for sidewalk versus road scenarios (Study 2).

contrast	estimate	SE	df	z	p	
road − sidewalk	0.8677	0.2576	Inf	3.369	.0008	***

Note: * $p < .05$, ** $p < .01$, *** $p < .001$.

Table S16: Estimated marginal means for acceptability of swerving in sidewalk versus road scenarios (Study 2).

scenario	EMM	SE	df	lower asymptotic 95% CI	upper asymptotic 95% CI
road	2.0196	0.3237	Inf	1.3852	2.6540
sidewalk	1.1519	0.3579	Inf	0.4504	1.8534

Table S17: Follow-up comparisons of lives-at-risk in pedestrians versus pedestrians scenario (Study 2).

contrast	odds ratio	SE	*df*	z lives-at-risk	p	
1v1 / 2v1	0.0206	0.0054	Inf	−14.706	<.0001	***
1v1 / 3v1	0.0061	0.0019	Inf	−16.347	<.0001	***
1v1 / 4v1	0.0043	0.0014	Inf	−16.739	<.0001	***
2v1 / 3v1	0.2950	0.0606	Inf	−5.940	<.0001	***
2v1 / 4v1	0.2092	0.0452	Inf	−7.241	<.0001	***
3v1 / 4v1	0.7089	0.1549	Inf	−1.574	.3933	

Note: * $p < .05$, ** $p < .01$, *** $p < .001$.

Table S18: Follow-up comparisons of lives-at-risk in pedestrians versus pedestrians scenario.

contrast	P(*swerve*)	SE	*df*	z lives-at-risk	p	
1v1 / 2v1	0.0206	0.0054	Inf	−14.706	<.0001	***
1v1 / 3v1	0.0061	0.0019	Inf	−16.347	<.0001	***
1v1 / 4v1	0.0043	0.0014	Inf	−16.739	<.0001	***
2v1 / 3v1	0.2950	0.0606	Inf	−5.940	<.0001	***
2v1 / 4v1	0.2092	0.0452	Inf	−7.241	<.0001	***
3v1 / 4v1	0.7089	0.1549	Inf	−1.574	.3933	

Note: * $p < .05$, ** $p < .01$, *** $p < .001$.

Table S19: Estimated marginal means for Lives-At-Risk factor in pedestrians versus pedestrians scenario (Study 2).

lives-at-risk	P(swerve)	SE	df	lower asymptotic 95% CI	upper asymptotic 95% CI
1v1	.1170	0.0346	Inf	0.0642	0.2036
2v1	.8653	0.0397	Inf	0.7672	0.9260
3v1	.9561	0.0153	Inf	0.9140	0.9780
4v1	.9685	0.0114	Inf	0.9365	0.9846

Table S20: Preference of swerving for lives-at-risk × perspective interaction in pedestrians versus pedestrians scenario

lives-at-risk	P ("swerve")	SE	df	lower asymptotic 95% CI	upper asymptotic 95% CI
perspective = car					
1v1	0.0668	0.0304	Inf	0.0268	0.1571
2v1	0.9474	0.0260	Inf	0.8664	0.9804
3v1	0.9854	0.0084	Inf	0.9554	0.9953
4v1	0.9809	0.0106	Inf	0.9441	0.9936
perspective = observer					
1v1	0.0771	0.0339	Inf	0.0317	0.1753
2v1	0.9152	0.0394	Inf	0.7997	0.9669
3v1	0.9565	0.0224	Inf	0.8846	0.9844
4v1	0.9705	0.0161	Inf	0.9161	0.9900
perspective = pedestrian fwd					
1v1	0.1561	0.0616	Inf	0.0688	0.3163
2v1	0.6126	0.1088	Inf	0.3917	0.7952
3v1	0.9523	0.0240	Inf	0.8761	0.9825
4v1	0.9671	0.0174	Inf	0.9098	0.9884
perspective = pedestrian side					
1v1	0.2178	0.0837	Inf	0.0961	0.4216
2v1	0.8468	0.0680	Inf	0.6643	0.9392
3v1	0.8837	0.0549	Inf	0.7272	0.9559
4v1	0.9472	0.0285	Inf	0.8543	0.9821

Table S21: Follow-up comparisons for lives-at-risk × perspective × motorist-type interaction in pedestrians versus pedestrians scenario.

contrast	odds ratio	SE	df	z lives-at-risk	p	
perspective = car occupant						
motorist = self-driving car						
1v1 / 2v1	0.0066	0.0043	Inf	−7.715	<.0001	***
1v1 / 3v1	0.0018	0.0013	Inf	−8.377	<.0001	***
1v1 / 4v1	0.0026	0.0019	Inf	−8.257	<.0001	***
2v1 / 3v1	0.2661	0.1588	Inf	−2.218	.1182	
2v1 / 4v1	0.3893	0.2216	Inf	−1.657	.3466	
3v1 / 4v1	1.4633	0.9111	Inf	0.611	.9285	
perspective = observer						
motorist = self-driving car						
1v1 / 2v1	0.0097	0.0057	Inf	−7.869	<.0001	***
1v1 / 3v1	0.0046	0.0030	Inf	−8.315	<.0001	***
1v1 / 4v1	0.0017	0.0013	Inf	−8.574	<.0001	***

contrast	odds ratio	SE	df	z lives-at-risk	p	
2v1 / 3v1	0.4685	0.2592	Inf	−1.370	.5180	
2v1 / 4v1	0.1747	0.1108	Inf	−2.752	.0302	*
3v1 / 4v1	0.3730	0.2421	Inf	−1.519	.4257	
perspective = pedestrian forward						
motorist = self-driving car						
1v1 / 2v1	0.0320	0.0188	Inf	−5.869	<.0001	***
1v1 / 3v1	0.0027	0.0020	Inf	−7.884	<.0001	***
1v1 / 4v1	0.0033	0.0024	Inf	−7.814	<.0001	***
2v1 / 3v1	0.0851	0.0518	Inf	−4.050	.0003	***
2v1 / 4v1	0.1040	0.0615	Inf	−3.830	.0007	***
3v1 / 4v1	1.2224	0.7812	Inf	0.314	.9893	
perspective = pedestrian side						
motorist = self-driving car						
1v1 / 2v1	0.0395	0.0228	Inf	−5.594	<.0001	***
1v1 / 3v1	0.0344	0.0202	Inf	−5.744	<.0001	***
1v1 / 4v1	0.0073	0.0051	Inf	−7.040	<.0001	***
2v1 / 3v1	0.8705	0.4623	Inf	−0.261	.9938	
2v1 / 4v1	0.1847	0.1140	Inf	−2.737	.0315	*
3v1 / 4v1	0.2122	0.1313	Inf	−2.506	.0590	
perspective = car occupant						
motorist = human						
1v1 / 2v1	0.0024	0.0016	Inf	−9.147	<.0001	***
1v1 / 3v1	0.0006	0.0005	Inf	−9.708	<.0001	***
1v1 / 4v1	0.0008	0.0006	Inf	−9.667	<.0001	***
2v1 / 3v1	0.2689	0.1532	Inf	−2.306	.0966	
2v1 / 4v1	0.3171	0.1762	Inf	−2.067	.1641	
3v1 / 4v1	1.1793	0.7115	Inf	0.273	.9929	
perspective = observer						
motorist = human						
1v1 / 2v1	0.0061	0.0040	Inf	−7.821	<.0001	***
1v1 / 3v1	0.0032	0.0022	Inf	−8.189	<.0001	***
1v1 / 4v1	0.0038	0.0026	Inf	−8.096	<.0001	***
2v1 / 3v1	0.5151	0.2996	Inf	−1.141	.6643	
2v1 / 4v1	0.6159	0.3528	Inf	−0.846	.8324	
3v1 / 4v1	1.1958	0.7140	Inf	0.299	.9907	
perspective = pedestrian forward						
motorist = human						
1v1 / 2v1	0.4276	0.1922	Inf	−1.890	.2324	
1v1 / 3v1	0.0316	0.0174	Inf	−6.277	<.0001	***
1v1 / 4v1	0.0119	0.0075	Inf	−7.036	<.0001	***
2v1 / 3v1	0.0738	0.0386	Inf	−4.981	<.0001	***
2v1 / 4v1	0.0279	0.0168	Inf	−5.943	<.0001	***

contrast	odds ratio	SE	df	z lives-at-risk	p	
3v1 / 4v1	0.3775	0.2199	Inf	-1.672	.3383	
perspective = pedestrian side						
motorist = human						
1v1 / 2v1	0.0642	0.0370	Inf	-4.768	<.0001	***
1v1 / 3v1	0.0390	0.0237	Inf	-5.339	<.0001	***
1v1 / 4v1	0.0330	0.0205	Inf	-5.503	<.0001	***
2v1 / 3v1	0.6081	0.3496	Inf	-0.865	.8229	
2v1 / 4v1	0.5148	0.3003	Inf	-1.138	.6657	
3v1 / 4v1	0.8465	0.5028	Inf	-0.281	.9923	

Note: * $p < .05$, ** $p < .01$, *** $p < .001$.

Table S23: Preference for swerving in pedestrian versus pedestrian scenario (Study 2).

lives-at-risk	P("swerve")	SE	df	lower asymptotic 95% CI	upper asymptotic 95% CI
perspective = car					
motorist = self-driving					
1v1	0.1275	0.0686	Inf	0.0418	0.3284
2v1	0.9569	0.0286	Inf	0.8506	0.9886
3v1	0.9881	0.0092	Inf	0.9472	0.9974
4v1	0.9827	0.0127	Inf	0.9288	0.9960
perspective = observer					
motorist = self-driving					
1v1	0.1089	0.0591	Inf	0.0357	0.2873
2v1	0.9262	0.0454	Inf	0.7736	0.9787
3v1	0.9640	0.0246	Inf	0.8695	0.9908
4v1	0.9863	0.0107	Inf	0.9387	0.9970
perspective = pedestrian (forward)					
motorist = self-driving					
1v1	0.1153	0.0637	Inf	0.0369	0.3072
2v1	0.8030	0.0981	Inf	0.5473	0.9322
3v1	0.9796	0.0151	Inf	0.9165	0.9952
4v1	0.9751	0.0178	Inf	0.9028	0.9940
perspective = pedestrian (side)					
motorist = self-driving					
1v1	0.1737	0.0922	Inf	0.0563	0.4255
2v1	0.8417	0.0912	Inf	0.5816	0.9532
3v1	0.8593	0.0838	Inf	0.6108	0.9597
4v1	0.9664	0.0252	Inf	0.8624	0.9925
perspective = car					
motorist = human					

lives-at-risk	P("swerve")	SE	df	lower asymptotic 95% CI	upper asymptotic 95% CI
1v1	0.0339	0.0207	Inf	0.0101	0.1080
2v1	0.9361	0.0384	Inf	0.8063	0.9810
3v1	0.9820	0.0128	Inf	0.9298	0.9956
4v1	0.9788	0.0147	Inf	0.9200	0.9946
perspective = observer					
motorist = human					
1v1	0.0540	0.0320	Inf	0.0165	0.1630
2v1	0.9028	0.0587	Inf	0.7145	0.9718
3v1	0.9475	0.0351	Inf	0.8188	0.9863
4v1	0.9378	0.0406	Inf	0.7940	0.9833
perspective = pedestrian (forward)					
motorist = human					
1v1	0.2078	0.1006	Inf	0.0734	0.4649
2v1	0.3802	0.1426	Inf	0.1579	0.6676
3v1	0.8926	0.0635	Inf	0.6941	0.9682
4v1	0.9565	0.0301	Inf	0.8419	0.9891
perspective = pedestrian (side)					
motorist = human					
1v1	0.2694	0.1261	Inf	0.0951	0.5641
2v1	0.8518	0.0868	Inf	0.5988	0.9568
3v1	0.9043	0.0612	Inf	0.7027	0.9742
4v1	0.9178	0.0541	Inf	0.7324	0.9785

Note: * $p < .05$, ** $p < .01$, *** $p < .001$.

Table S25: Follow-up comparisons for lives-at-risk × perspective interaction for pedestrians versus pedestrians scenario.

contrast	odds ratio	SE	df	z lives-at-risk	p	
lives-at-risk= 1v1						
car occupant / observer	0.8579	0.4594	Inf	−0.286	.9918	
car occupant / pedestrian (in car's path)	0.3873	0.2149	Inf	−1.710	.3186	
car occupant / pedestrian (to side of car's path)	0.2573	0.1505	Inf	−2.320	.0935	
observer / pedestrian (in car's path)	0.4514	0.2491	Inf	−1.441	.4735	
observer / pedestrian (to side of car's path)	0.2999	0.1748	Inf	−2.066	.1644	
pedestrian (in car's path) / pedestrian (to side of car's path)	0.6643	0.3761	Inf	−0.722	.8882	
lives-at-risk = 2v1						
car occupant / observer	1.6701	0.9872	Inf	0.868	.8216	
car occupant / pedestrian (in car's path)	11.3976	6.7101	Inf	4.133	.0002	***
car occupant / pedestrian (to side of car's path)	3.2605	2.0813	Inf	1.852	.2493	
observer / pedestrian (in car's path)	6.8247	3.9824	Inf	3.291	.0055	**
observer / pedestrian (to side of car's path)	1.9523	1.2394	Inf	1.054	.7176	
pedestrian (in car's path) / pedestrian (to side of car's path)	0.2861	0.1710	Inf	−2.094	.1550	
lives-at-risk = 3v1						
car / observer	3.0675	2.0122	Inf	1.709	.3190	
car / pedestrian (in car's path)	3.3778	2.2723	Inf	1.809	.2688	
car / pedestrian (to side of car's path)	8.8704	6.1554	Inf	3.145	.0090	**
observer / pedestrian (in car's path)	1.1011	0.7140	Inf	0.149	.9988	
observer / pedestrian (to side of car's path)	2.8917	1.9156	Inf	1.603	.3769	
pedestrian (in car's path) / pedestrian (to side of car's path)	2.6261	1.7060	Inf	1.486	.4458	
lives-at-risk = 4v1						
car / observer	1.5594	1.0308	Inf	0.672	.9077	
car / pedestrian (in car's path)	1.7467	1.1731	Inf	0.830	.8400	
car / pedestrian (to side of car's path)	2.8616	2.0073	Inf	1.499	.4381	
observer / pedestrian (in car's path)	1.1201	0.7601	Inf	0.167	.9983	
observer / pedestrian (to side of car's path)	1.8351	1.2945	Inf	0.861	.8251	
pedestrian (in car's path) / pedestrian (to side of car's path)	1.6383	1.1199	Inf	0.722	.8883	

Note: * $p < .05$, ** $p < .01$, *** $p < .001$.

Table S26: Follow-up comparisons for lives-at-risk in car occupants versus pedestrians scenario (Study 2).

contrast	odds ratio	SE	df	z lives-at-risk	p	
1v1 / 2v1	0.2037	0.0673	Inf	-4.813	$< .0001$	***
1v1 / 3v1	0.0540	0.0220	Inf	-7.164	$< .0001$	***
1v1 / 4v1	0.0299	0.0133	Inf	-7.871	$< .0001$	***
2v1 / 3v1	0.2650	0.0924	Inf	-3.807	.0008	***
2v1 / 4v1	0.1468	0.0559	Inf	-5.038	<.0001	***
3v1 / 4v1	0.5541	0.2037	Inf	-1.606	.3751	

Note: * $p < .05$, ** $p < .01$, *** $p < .001$.

Table S27: Preference of swerving for different lives-at-risk in car occupants versus pedestrians scenario (Study 2).

lives-at-risk	P(swerve)	SE	df	lower asymptotic 95% CI	upper asymptotic 95% CI
1v1	0.3442	0.1330	Inf	0.1419	0.6249
2v1	0.7204	0.1215	Inf	0.4413	0.8937
3v1	0.9067	0.0541	Inf	0.7349	0.9715
4v1	0.9461	0.0337	Inf	0.8276	0.9847

Table S28: Follow up comparison of motorist-type × Lives-At-Risk factor interaction (Study 2).

contrast	odds ratio	SE	df	z	p	
motorist = self-driving						
2v1 / 1v1	9.5327	4.5049	Inf	4.771	<.0001	***
3v1 / 1v1	35.3768	19.8466	Inf	6.357	<.0001	***
4v1 / 1v1	50.2008	30.0540	Inf	6.541	<.0001	***
motorist = human						
2v1 / 1v1	2.1436	0.8766	Inf	1.865	0.1578	
3v1 / 1v1	6.6640	3.0346	Inf	4.165	0.0001	***
4v1 / 1v1	16.1888	8.2770	Inf	5.446	<.0001	***

Note: * $p < .05$, ** $p < .01$, *** $p < .001$.

Table S29: Preference for swerving based on motorist-type (Study 2).

lives-at-risk	P("swerve")	SE	df	lower asymptotic 95% CI	upper asymptotic 95% CI
motorist = self-driving					
1v1	0.2913	0.1331	Inf	0.1041	0.5924
2v1	0.7967	0.1085	Inf	0.5131	0.9358
3v1	0.9357	0.0435	Inf	0.7794	0.9836
4v1	0.9538	0.0329	Inf	0.8267	0.9889
motorist = human					
1v1	0.4308	0.1621	Inf	0.1717	0.7344
2v1	0.6187	0.1570	Inf	0.3058	0.8567
3v1	0.8346	0.0953	Inf	0.5659	0.9513
4v1	0.9246	0.0506	Inf	0.7475	0.9807

Table S30: Follow up comparisons for lives-at-risk × perspective× motorist-type in pedestrian versus car occupant scenario (Study 2).

contrast	odds ratio	SE	df	z	p	
perspective = car						
motorist = self-driving						
2v1 / 1v1	8.2638	6.6516	Inf	2.624	.0244	*
3v1 / 1v1	15.8926	13.7236	Inf	3.203	.0040	**
4v1 / 1v1	33.9867	32.2364	Inf	3.717	.0006	***
perspective = observer						
motorist = self-driving						
2v1 / 1v1	9.1710	7.1300	Inf	2.850	.0124	
3v1 / 1v1	35.0391	31.8736	Inf	3.910	.0003	***
4v1 / 1v1	12.3135	9.8459	Inf	3.140	.0049	**
perspective = pedestrian						
motorist = self-driving						
2v1 / 1v1	14.8127	12.6942	Inf	3.145	.0048	**
3v1 / 1v1	101.2472	104.1660	Inf	4.488	<.0001	***
4v1 / 1v1	462.8984	576.8877	Inf	4.925	<.0001	***
perspective = car						
motorist = human						
2v1 / 1v1	5.7257	4.3716	Inf	2.285	.0604	
3v1 / 1v1	68.0244	69.9305	Inf	4.105	.0001	***
4v1 / 1v1	29.5189	27.3060	Inf	3.659	.0007	***
perspective = observer						
motorist = human						
2v1 / 1v1	2.7827	2.0350	Inf	1.399	.3644	
3v1 / 1v1	3.6255	2.6863	Inf	1.738	.2029	
4v1 / 1v1	20.4203	17.2913	Inf	3.562	.0011	**
perspective = pedestrian						
motorist = human						
2v1 / 1v1	0.7835	0.5459	Inf	−0.350	.9496	
3v1 / 1v1	2.9083	2.1650	Inf	1.434	.3453	
4v1 / 1v1	11.9686	10.5255	Inf	2.823	.0136	*

Note: * $p < .05$, ** $p < .01$, *** $p < .001$.

Table S31: Estimated marginal means for lives-at-risk × perspective × motorist-type interaction in car occupants versus pedestrians scenario

contrast	odds ratio	SE	df	z lives-at-risk	p	
perspective = car occupant						
motorist = self-driving car						
1v1 / 2v1	0.1210	0.0974	Inf	−2.624	.0432	*
1v1 / 3v1	0.0629	0.0543	Inf	−3.203	.0074	**
1v1 / 4v1	0.0294	0.0279	Inf	−3.717	.0012	**
2v1 / 3v1	0.5200	0.4258	Inf	−0.799	.8551	
2v1 / 4v1	0.2431	0.2131	Inf	−1.613	.3710	
3v1 / 4v1	0.4676	0.4111	Inf	−0.865	.8231	
perspective = observer						
motorist = self-driving car						
1v1 / 2v1	0.1090	0.0848	Inf	−2.850	.0227	*
1v1 / 3v1	0.0285	0.0260	Inf	−3.910	.0005	***
1v1 / 4v1	0.0812	0.0649	Inf	−3.140	.0092	**
2v1 / 3v1	0.2617	0.2225	Inf	−1.577	.3918	
2v1 / 4v1	0.7448	0.5733	Inf	−0.383	.9809	
3v1 / 4v1	2.8456	2.4293	Inf	1.225	.6109	
perspective = pedestrian						
motorist = self-driving car						
1v1 / 2v1	0.0675	0.0579	Inf	−3.145	.0090	**
1v1 / 3v1	0.0099	0.0102	Inf	−4.488	<.0001	***
1v1 / 4v1	0.0022	0.0027	Inf	−4.925	<.0001	***
2v1 / 3v1	0.1463	0.1281	Inf	−2.195	.1245	
2v1 / 4v1	0.0320	0.0350	Inf	−3.149	.0089	**
3v1 / 4v1	0.2187	0.2310	Inf	−1.439	.4750	
perspective = car occupant						
motorist = human						
1v1 / 2v1	0.1747	0.1333	Inf	−2.285	.1014	
1v1 / 3v1	0.0147	0.0151	Inf	−4.105	.0002	***
1v1 / 4v1	0.0339	0.0313	Inf	−3.659	.0014	
2v1 / 3v1	0.0842	0.0801	Inf	−2.602	.0458	
2v1 / 4v1	0.1940	0.1673	Inf	−1.902	.2272	
3v1 / 4v1	2.3044	2.1254	Inf	0.905	.8021	
perspective = observer						
motorist = human						
1v1 / 2v1	0.3594	0.2628	Inf	−1.399	.4997	
1v1 / 3v1	0.2758	0.2044	Inf	−1.738	.3038	
1v1 / 4v1	0.0490	0.0415	Inf	−3.562	.0021	**
2v1 / 3v1	0.7675	0.5581	Inf	−0.364	.9835	
2v1 / 4v1	0.1363	0.1096	Inf	−2.479	.0632	
3v1 / 4v1	0.1775	0.1413	Inf	−2.172	.1309	
perspective = pedestrian						
motorist = human						
1v1 / 2v1	1.2763	0.8892	Inf	0.350	.9853	
1v1 / 3v1	0.3438	0.2560	Inf	−1.434	.4780	
1v1 / 4v1	0.0836	0.0735	Inf	−2.823	.0246	*
2v1 / 3v1	0.2694	0.2009	Inf	−1.758	.2936	
2v1 / 4v1	0.0655	0.0580	Inf	−3.078	.0112	*
3v1 / 4v1	0.2430	0.2127	Inf	−1.616	.3694	

Note: * $p < .05$, ** $p < .01$, *** $p < .001$.

Table S32: Preference for swerving for lives-at-risk × perspective × motorist-type interaction in car occupants versus pedestrians scenario.

lives-at-risk	$P(\text{swerve})$	SE	df	lower asymptotic 95% CI	upper asymptotic 95% CI
perspective = car occupant					
motorist = self-driving car					
1v1	0.3681	0.2336	Inf	0.0752	0.8065
2v1	0.8280	0.1496	Inf	0.3804	0.9742
3v1	0.9025	0.0954	Inf	0.5249	0.9873
4v1	0.9519	0.0521	Inf	0.6798	0.9946
perspective = observer					
motorist = self-driving car					
1v1	0.4146	0.2438	Inf	0.0900	0.8353
2v1	0.8666	0.1220	Inf	0.4509	0.9809
3v1	0.9613	0.0424	Inf	0.7271	0.9957
4v1	0.8971	0.0986	Inf	0.5179	0.9861
perspective = pedestrian					
motorist = self-driving car					
1v1	0.1614	0.1338	Inf	0.0270	0.5720
2v1	0.7403	0.1900	Inf	0.2913	0.9518
3v1	0.9512	0.0504	Inf	0.6987	0.9939
4v1	0.9889	0.0137	Inf	0.8856	0.9990
perspective = car occupant					
motorist = human					
1v1	0.3694	0.2323	Inf	0.0766	0.8053
2v1	0.7704	0.1824	Inf	0.3077	0.9620
3v1	0.9755	0.0285	Inf	0.7939	0.9976
4v1	0.9453	0.0580	Inf	0.6569	0.9936
perspective = observer					
motorist = human					
1v1	0.1715	0.1428	Inf	0.0281	0.5973
2v1	0.3656	0.2305	Inf	0.0759	0.8016
3v1	0.4288	0.2436	Inf	0.0965	0.8406
4v1	0.8087	0.1601	Inf	0.3574	0.9698
perspective = pedestrian					
motorist = human					
1v1	0.6844	0.2191	Inf	0.2289	0.9406
2v1	0.6295	0.2357	Inf	0.1899	0.9249
3v1	0.8632	0.1238	Inf	0.4470	0.9801
4v1	0.9629	0.0408	Inf	0.7347	0.9959
